# Systematic Review of Inhaled Bronchodilator and Corticosteroid Therapies in Infants with Bronchopulmonary Dysplasia: Implications and Future Directions

**DOI:** 10.1371/journal.pone.0148188

**Published:** 2016-02-03

**Authors:** Brian J. Clouse, Sudarshan R. Jadcherla, Jonathan L. Slaughter

**Affiliations:** 1 Center for Perinatal Research, The Research Institute at Nationwide Children’s Hospital, Columbus, Ohio, United States of America; 2 Division of Neonatology, Department of Pediatrics, Nationwide Children’s Hospital, Columbus, Ohio, United States of America; 3 The Ohio State University College of Medicine, Columbus, Ohio, United States of America; Technion - Israel Institute of Technology, ISRAEL

## Abstract

**Background:**

There is much debate surrounding the use of inhaled bronchodilators and corticosteroids for infants with bronchopulmonary dysplasia (BPD).

**Objective:**

The objective of this systematic review was to identify strengths and knowledge gaps in the literature regarding inhaled therapies in BPD and guide future research to improve long-termoutcomes.

**Methods:**

The databases of Academic Search Complete, CINAHL, PUBMED/MEDLINE, and Scopus were searched for studies that evaluated both acute and long-term clinical outcomes related to the delivery and therapeutic efficacy of inhaled beta-agonists, anticholinergics and corticosteroids in infants with developing and/or established BPD.

**Results:**

Of 181 articles, 22 met inclusion criteria for review. Five evaluated beta-agonist therapies (*n = 84*, weighted gestational age (GA) of 27.1(26–30) weeks, weighted birth weight (BW) of 974(843–1310) grams, weighted post menstrual age (PMA) of 34.8(28–39) weeks, and weighted age of 53(15–86) days old at the time of evaluation). Fourteen evaluated inhaled corticosteroids (*n = 2383*, GA 26.2(26–29) weeks, weighted BW of 853(760–1114) grams, weighted PMA of 27.0(26–31) weeks, and weighted age of 6(0–45) days old at time of evaluation). Three evaluated combination therapies (*n = 198*, weighted GA of 27.8(27–29) weeks, weighted BW of 1057(898–1247) grams, weighted PMA of 30.7(29–45) weeks, and age 20(10–111) days old at time of evaluation).

**Conclusion:**

Whether inhaled bronchodilators and inhaled corticosteroids improve long-term outcomes in BPD remains unclear. Literature regarding these therapies mostly addresses evolving BPD. There appears to be heterogeneity in treatment responses, and may be related to varying modes of administration. Further research is needed to evaluate inhaled therapies in infants with severe BPD. Such investigations should focus on appropriate definitions of disease and subject selection, timing of therapies, and new drugs, devices and delivery methods as compared to traditional methods across all modalities of respiratory support, in addition to the assessment of long-term outcomes of initial responders.

## Introduction

Many pharmacological interventions are used to treat BPD in preterm infants with the aim of optimizing pulmonary function as well as reducing concomitant co-morbidities [1–4]. The chronic pulmonary insufficiency associated with BPD often has a deleterious effect on the normal development of multiple organ systems, making the overall illness difficult to manage [5, 6].

Inhaled medications such as beta-agonists, anticholinergics and corticosteroids (ICS) have been used to treat airway disease in infants with BPD. Beta-agonists and anticholinergics and have been shown to acutely improve pulmonary function. However, the prescription of such therapies remains highly variable due to insufficient evidence to support improved long-term outcomes [6–12]. Several heterogeneous factors such as disease severity, variation in response among subjects, differences in aerosol delivery methods, choice of therapeutic agents, dose prescribed or actual dose delivered to the targeted site could potentially affect the interpretation of clinical outcomes. The purpose of this critical review is to identify therapeutic effect-influencing factors among existing randomized control trials that assessed inhaled bronchodilator and corticosteroid use among infants with BPD, so as to guide future research protocols aimed to improve long-term outcomes.

## Methods

### Literature Search

The databases of Academic Search Complete, CINAHL, PUBMED/MEDLINE, and Scopus were searched using the Boolean phrase: ((bronchopulmonary dysplasia AND (inhaled bronchodilators OR inhaled beta agonists OR inhaled corticosteroids OR inhaled anticholinergics NOT nitric oxide))). The databases were searched from their earliest indexed dates to December 1, 2015. The reference lists of included manuscripts were reviewed in search of sources that may have been missed.

### Selection Criteria

A search filter was applied to limit results to include studies of randomized controlled design. Two reviewers (BJC and JLS) independently appraised the literature. Completed studies with a purpose of assessing delivery, efficacy, and clinical outcomes of beta-agonists, anticholinergics and inhaled corticosteroids (ICS) in infants with developing and/or established BPD (as evident by chest radiograph [13] or current definition [14]) were selected for inclusion in this review.

### Data Collection and Appraisal

The guidelines of the PRISMA Statement for presentation of systematic reviews were followed to report the findings of this systematic review (see S1 File) [15]. The Matrix Method was used to organize, extract, and appraise data compiled from included studies [16]. Specific data extracted included subject demographics, inhaled drug classifications, dosages, delivery methods, aerosol characteristics, outcome measures (short-term vs. long-term), and results. Short-term outcomes were defined as a subject’s response to a single treatment session, whereas long-term outcomes were defined a subject’s response to multiple treatment sessions.

The methodological quality of the data was appraised for potential biases. Details pertaining to randomization (listed method or unclear), allocation concealment (listed method or unclear), double blinding (yes, no, or unclear), power calculation (yes or no) and reporting of exact point statistical methods (yes or no) were collected. Each study [17–38] was scored for three levels of potential bias (low, high, or unclear). A “no” answer in any category was scored as a high risk for potential bias, whereas one “unclear” with otherwise all “yes” answers was scored as an unclear risk of bias. In order to classified as a low risk of potential bias, all methods assessed had to be clear, the study had to be double blinded, a power calculation performed, and exact point statistics used.

## Results

**Fig 1:**
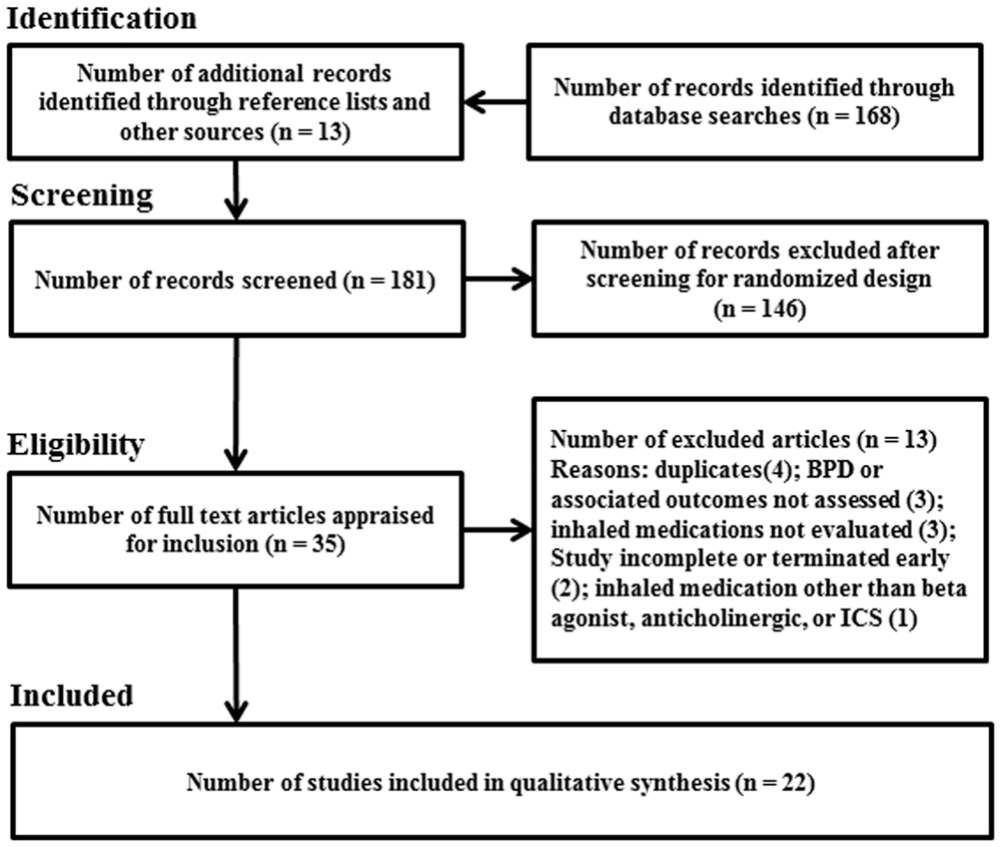
PRISMA Flow Diagram. Flow diagram of the systematic review revealing the pathway to the included studies.

Database searches with the Boolean phrase (as described above) yielded 168 results. Thirteen additional studies were identified from the reference lists of select articles. A total of 181 articles were screened. After filtering to exclude studies of non-randomized controlled design, thirty-five articles remained. Thirteen articles were excluded during appraisal (4 duplicates, 3 did not focus on BPD, 3 did not evaluate inhaled medications, 2 were incomplete, and 1 evaluated inhaled medications other than bronchodilators, or ICS), leaving 22 articles included in the review (Fig 1). Characteristics of the 22 included studies [17–38] can be found in Table 1. Quality assessment of the data revealed an overall high risk for potential bias in the included studies as shown in Table 2.

**Table 1:** Characteristics of Included Randomized Control Trials (*n = 22*).

First Author (year)	Inclusion Criteria*	n	GA† (wk)	BW† (g)	Age† (d)	Inhaled Medication	Delivery Device/Dose‡	MDI Particle Size (MMAD)	Resp. Support	Outcome Type§	Outcome Measures	Summary of Findings
*Inhaled Beta-agonist*s												
Fok[17] 1996	age>2wk, BPD, on resp. support during first wk of life	23	28	1097	82	salbutamol	jet neb, 100μg/kg, MDI(CFC) 200μg	2.3μm	mech. vent. & spont. breathing	short-term	gamma imaging	aerosol deposition small and variable MDI delivery > jet neb.
Fok[18] 1998	GA<32 wk, BW<1500g, age>2wk, BPD on resp. support during first week of life	20	27	843	51	salbutamol	MDI(CFC), ultrasonic neb., jet neb., 200μg	2.3μm	mech. vent.	short-term	HR, TCCO2, TCO2, Crs, Rrs	aerosol delivery order of efficiency: ultrasonic>MDI>jet neb.
Gappa[19] 1997	responsive to inhaled therapies or established BPD	13	27	903	69	salbutamol	jet neb., 600μg, MDI(CFC) 200μg	2.3μm	spont. breathing	short-term	C_L,dyn_, R_L,dyn_	inhaled salbutamol improves lung mechanics with jet neb. & MDI being equally efficient
Pfenninger[20] 1993	BPD, on mech. vent.	8	30	1310	48	salbutamol	MDI(CFC) 200μg, IV, 10μg/kg	2.3μm	mech. vent.	short-term	HR, BP, CBG, TCO2, Crs, Rrs	inhaled & IV salbutamol improves lung mechanics equally with comparable cardiac side effects
Rotschild[21] 1989	GA = preterm, BW<1500, BPD on mech. vent.	20	26	875	15	salbutamol	jet neb., 2.5mg	NA	mech. vent.	short-term	HR, BP, PO2, PCO2, Crs, Rrs	early bronchodilator therapy may be beneficial in preterm infants with BPD
*Inhaled Corticosteroids*												
Bassler[22] 2015	GA ≥23 <28wk, age≤12h, on positive pressure	863	26	801	0	budesonide	MDI(HFA), 800μg/d x 14d, then 400μg/d from 15d to 32 wk PMA	3.9μm	mech. vent. & spont. breathing	long-term	death or BPD at 36wk PMA	incidence of BPD was less in treatment group vs placebo, budesonide may increase mortality
Cole[23] 1999	GA<33wk, BW<1251g, age 3–14d on mech. vent.	253	26	801	6	beclometh.	MDI(CFC), 1000μg/kg/d 4wk taper to 125μg/kg/d	3.5μm	mech. vent.	long-term	BPD	early ICS in infants at risk for BPD was associated with less subsequent systemic steroid use, bronchodilator use, & mech. vent. on DOL 28
Cole[24] 1999	GA<33wk, BW<1251g, age 3–14d.	148	26	838	27	beclometh.	MDI(CFC), 1000μg/kg/d 4wk taper to 125μg/kg/d	3.5μm	mech. vent.	long-term	plasma cortisol levels	ICS was associated with mildly reduced plasma cortisol levels, no adrenal suppression on cosyntropin stimulation
Dimitriou[25] 1997	GA<32wk, on mech. vent. = 5d or suppl. O2 = 14d BW<1251g, age 3–14d.	40	27	834	27	budesonide	MDI(CFC), 400μg/d	4.0μm	mech. vent. & spont. breathing	long-term	duration of resp. support, Crs, BP, adverse events	systemic steroids have a faster onset of action versus ICS
Dugas[26] 2005	GA<32wk, age 28–60d, BPD, on resp. support	32	27	961	45	fluticasone	MDI(CFC), 250μg/d x 3wk, 125μg/d x 1wk||	2.6μm	mech. vent. & spont. breathing	long-term	duration of O2, survival with O2	fluticasone does not reduce O2 need in infants with moderate BPD
Fok[27] 1999	GA<32wk, BW<1500g, age = 1d, RDS, on mech. vent	53	28	987	1	fluticasone	MDI(CFC), 1000μg/d x 2wk	2.6μm	mech. vent	long-term	extubation at 7&14d, Crs, Rrs, death, BPD	more cases versus controls extubated on DOL 14 & had increased Crs, no difference in need for systemic steroids or the development of BPD
Merz[28] 1999	GA = 25–32wk, BW = 750–1500g, age = 3d, on mech. vent.	23	29	1114	3	budesonide	MDI(CFC), 1600μg/d x 10d or extubation	4.5μm	mech. vent	long-term	duration of resp. support, BPD, inflammatory markers, endocrine funct.	no outcome differences between cases & controls including side effects
Giep[29] 1996	BW<1500g, age≥2wk, RDS or evolving BPD, on mech. vent.	19	26	768	5	beclometh.	MDI(CFC), 1mg/kg/d	3.5μm	mech. vent	long-term	BP, HR, resp. support, cortisol levels, etc.	inhaled beclometh. can be safely delivered to intubated neonates
Gupta[30] 2000	GA<33wk, BW≤ 1250g, age 3–14d, on mech. vent.	161	26	794	5	beclometh.	MDI(CFC), 1000μg/kg/d 4wk taper to 125μg/kg/d	3.5μm	mech. vent	long-term	tracheal aspirates IL-8, IL-1ra	being a case was associated with decreased inflammation following 1 week of therapy, less systemic steroid use, & less incidence of BPD
Halliday[31] 2001	GA<30wk, age<72hr and/or>15d on mech. vent.	570	27	1007	2	budesonide	MDI(CFC), 800μg/kg/d x 12d	4.5μm	mech. vent.	long-term	death, O2 at 36wk PMA, duration of O2, adverse events	inhaled budesonide may be safer but no clear evidence regarding effectiveness versus dexamethasone
Kovács[32] 1998	GA<30, BW≤1500g, age = 7d	60	26	764	7	budesonide	jet neb. 1000μg/d x 18d	NA	mech. vent.	long-term	incidence of BPD	ICS not as effective as systemic steroids & do not improve BPD outcomes
LaForce[33] 1993	BW<1500g, age = 14d, RDS or BPD on mech. vent.	13	ND	ND	14	beclometh.	jet neb. 150μg/d x 28d	NA	mech. vent. & spont. breathing	long-term	Crs, Rrs, tracheal aspirate, blood/urin/CSF cultures	cases showed improved pulmonary mechanics following 3weeks of therapy
Rozycki[34] 2003	age = 14d at risk for BPD on mech. vent	61	26	760	14	beclometh.	MDI(CFC), 3 dose ranges, 17–129μg/kg/d	3.5μm	mech. vent.	long-term	extubation within 7d	low dose ICS was less effective than systemic steroids to facilitate extubation
Suchomski[35] 2002	GA<30wk, BW≤1500g, age = 12–21d	78	26	844	17	beclometh.	MDI(CFC), 2 dose levels 400 and 800μg/kg/d	3.5μm	mech. vent.	long-term	changes in vent settings, duration of resp support, BPD, LOS, adverse events	with delayed onset ICSs offer no advantage over systemic steroids and have similar risks
*Combination Therapies*												
Denjean[36] 1998	GA<31wk, age = 10d, RDS, on mech. vent.	173	28	1050	10	salbutamol & beclometh.	MDI(CFC), 1200μg/d & 1000μg/d taper over 8d	2.3μm & 3.5μm	mech. vent. & spont. breathing	long-term	BPD or death	no significant outcome differences among groups
Kao[37] 1989	BPD	15	29	1247	111	metaproterenol & atropine	jet neb., 1mg/kg & 0.8mg/kg	NA	spont. breathing	short-term	RR, Raw, Crs, VmaxFRC, TGV	metaproterenol and atropine independently had a positive effect on lung mechanics, however there were no synergistic effects
Kugelman[38] 2006	GA = preterm, spont. breathing with evolving BPD on inhaled medications	10	27	898	66	terbutaline & budesonide	jet neb., 2mg & 0.5ml	NA	spont. breathing	short-term	infant tolerance, user choice, respir. scores, HR, RR, SPO2, FIO2	hood therapy took less time, was better tolerated and as effective as mask therapy

*common exclusion criteria included but was not limited to congenital anomalies, pulmonary infection, sepsis, treatment with corticosteroids and bronchodilators, air leak disorders, etc.

^†^combined value from cases & controls, in some cases estimated based on reported data,

^‡^estimated dose emitted from canister for all MDI delivery methods,

^¶^ MMAD emitted from MDI actuator,values derived from references [39–41],

^§^ short-term outcome = single dose response, long-term outcome = outcome based on multiple treatment sessions (e.g. time to extubation, duration of therapy, development of BPD, length of stay, death, etc), || dose doubled if greater than 1200g

Table 1 abbreviations. beclomethasone (beclometh.), blood pressure (BP), bronchopulmonary dysplasia (BPD), birth weight (BW), capillary blood gas (CBG), lung compliance (C_L,dyn_), chlorofluorocarbon propellant (CFC), respiratory system compliance (Crs), cerebrospinal fluid (CSF), day (d), days of life (DOL), fraction of inspired oxygen (FIO2), gestational age (GA), hour (h), hydrofluoroalkane propellant (HFA), heart rate (HR), interleukin -1 receptor agonist (Il-1ra), interleukin-8 (IL-8), intravenous (IV), length of stay (LOS), minute (min), mechanical ventilation (mech. vent.), mass median aerodynamic diameter (MMAD), not applicable (NA), not defined (ND), nebulizer (neb.), partial pressure of carbon dioxide (PCO2), post menstrual age (PMA), partial pressure of oxygen (PO2), airway resistance (Raw), respiratory distress syndrome (RDS), lung resistance (R_L,dyn_), respiratory (resp.) respiratory rate (RR), respiratory system resistance (Rrs), seconds (s), oxygen saturation by pulse oximetry (SPO2), supplemental (suppl.), thoracic gas volume (TGV), treatment (TX), valved holding chamber (VHC), maximum exhaled volume from functional residual capacity (VmaxFRC)

**Table 2:** Quality Assessment of Included Randomized Controlled Trials *(n = 22)*.

First author (year)	Random sequence generation	Allocation concealment	Double Blinded	Power calculation	Exact point statistic reported	Risk of bias
Fok [17] 1996	Computer	Unclear	Unclear	No	No	High
Fok [18] 1998	Computer	Unclear	No	No	No	High
Gappa [19] 1997	Unclear	Unclear	Unclear	Yes	Yes (mean,CI)	Unclear
Pfenninger [20] 1993	Unclear	Envelope	Unclear	No	No	High
Rotschild [21] 1989	Unclear	Unclear	Yes	No	No	High
Bassler [22] 2015	Computer	Envelope	Yes	Yes	Yes (OR,CI)	Low
Cole [23] 1999	3rd party	Unclear	Yes	No	Yes (RR,CI)	High
Cole [24] 1999	3rd party	Unclear	Yes	No	No	High
Dimitriou [25] 1997	Unclear	Envelope	Unclear	Yes	No	High
Dugas [26] 2005	Block	Unclear	Yes	Yes	No	High
Fok [27] 1999	Computer	Envelope	No	Yes	Yes (OR,CI)	High
Merz [28] 1999	3rd party	Unclear	Yes	No	No	High
Giep [29] 1996	Unclear	Unclear	Yes	No	No	High
Gupta [30] 2000	3rd party	Unclear	Yes	No	No	High
Halliday [31] 2001	3rd party	Unclear	No	Yes	Yes (OR,CI)	High
Kovacs [32] 1998	Block	Unclear	Yes	Yes	No	High
LaForce [33] 1993	Unclear	Unclear	No	No	No	High
Rozycki [34] 2003	Number Table	Unclear	Yes	Yes	No	High
Suchomski [35] 2002	Unclear	Envelope	No	Yes	No	High
Denjean [36] 1998	Unclear	Unclear	Yes	Yes	Yes (OR,CI)	Unclear
Kao [37] 1989	Unclear	Unclear	Yes	No	No	High
Kugelman [38] 2006	Unclear	Envelope	No	Yes	No	High

### Inhaled Beta-Agonists

Five studies evaluated inhaled beta-agonist therapy with salbutamol (International Nonproprietary Name), also known as albuterol (United States Adopted Name) [17–21]. The combined characteristics of these studies are summarized in Table 3. These studies included 84 subjects with a weighted gestational age (GA) of 27.1 (26–30) weeks, weighted birth weight (BW) of 974 (843–1310) grams, weighted postmenstrual age (PMA) of 34.8 (28–39) weeks, and a weighted age of 53 (15–86) days old at the time of evaluation. Doses of 200 μg by metered dose inhaler (MDI) and 100μg/kg to 2.5 mg nebulized solution were used. Delivery methods included metered dose inhaler MDI, jet nebulizer, and ultrasonic nebulizer to both mechanically ventilated and spontaneously breathing subjects.

**Table 3:** Summary of Subject and Study Characteristics of 22 Randomized Control Trials of Inhaled Therapies in Bronchopulmonary Dysplasia.

	Beta-agonists	Inhaled Corticosteroids	Combination Therapies
Number of randomized control trials	5	14	3
*Subject Characteristics*			
Total number of subjects (n)	84	2383	198
Subjects who were treated (n)	84	1212	155
Gestational age*, range (weeks)	27.1(26–30)	26.2(26–29)	27.8(27–29)
Birth weight*, range (g)	974(843–1310)	853(760–1114)	1057(898–1247)
Post menstrual age at time of study* (weeks)	34.8(28–39)	27.0(26–31)	30.7(29–45)
Chronological age at time of study*, range (d)	53(15–86)	6(0–45)	20(10–111)
Duration of therapy*, range (d)	7(1–28)	27(7–56)	25(1–28)
Mechanically Ventilated (%)	76.2	86.4	57.6
*Delivery Devices*			
MDI (%)	40	96.3	87.4
Jet Nebulizer (%)	47.5	3.4	12.6
Ultra Sonic Nebulizer (%)	12.5	0	0
*Outcomes Assessed*			
Short-term (%)†	100	0	12.6
Long-term (%)‡	0	100	87.4

* Weighted average

^†^ Short-term outcome = single dose response

^‡^ Long-term outcome = outcome based on multiple treatment sessions (e.g. time to extubation, duration of therapy, development of BPD, length of stay, death, etc)

#### Beta-agonist delivery

Salbutamol delivery devices were evaluated in 3 of the 5 included beta-agonist studies [17–19]. Methods to assess delivery included radio-labeled aerosol with gamma imaging,[17] measurements of respiratory system compliance (Crs) and resistance (Rrs),[18] and measurements of lung compliance (C_L,dyn_) and resistance (R_L,dyn_) [19]. One study compared MDI and jet nebulizer efficacy in both spontaneously breathing and mechanically ventilated infants and concluded that larger doses, as measured by radio-labeled aerosol deposition, were delivered to both subject groups by MDI and holding chamber [17]. However, a similar study limited to spontaneously breathing subjects with BPD at 36 weeks postmenstrual age concluded there were no differences in delivery efficacy, measured as lung compliance and resistance, between the two modalities [19]. In the third study, an ultrasonic nebulizer, MDI, and jet nebulizer were compared in mechanically ventilated subjects [18]. Deposition to the periphery as determined by physiologic changes seemed to be greatest with an ultrasonic nebulizer placed 20 cm from the endotracheal tube, followed by the MDI with holding chamber, then the jet nebulizer respectively [18]. Studies assessing delivery of inhaled salbutamol demonstrate that aerosol delivery to the lower airways in both spontaneously breathing and mechanically ventilated infants is highly variable [17–19].

#### Short-term beta-agonist efficacy

The short-term efficacy of inhaled salbutamol was assessed in 2 of the 5 beta-agonist studies [20, 21]. The first study evaluated inhaled salbutamol MDI in comparison to systemic delivery by the IV route in mechanically ventilated subjects [20]. Analysis of Crs and Rrs following drug delivery revealed that both methods were equally efficacious showing Crs and Rrs improvements; both methods had a similar effect on heart rate. The second study assessed the efficacy of inhaled salbutamol by jet nebulizer compared to an inhaled placebo in mechanically ventilated preterm infants with evolving BPD [21]. Changes in partial pressure of carbon dioxide, Crs and Rrs following delivery were evaluated. This study concluded that inhaled beta agonists started as early as the second week of life may be beneficial in the treatment of evolving BPD.

### Inhaled Corticosteroids

**Fig 2:**
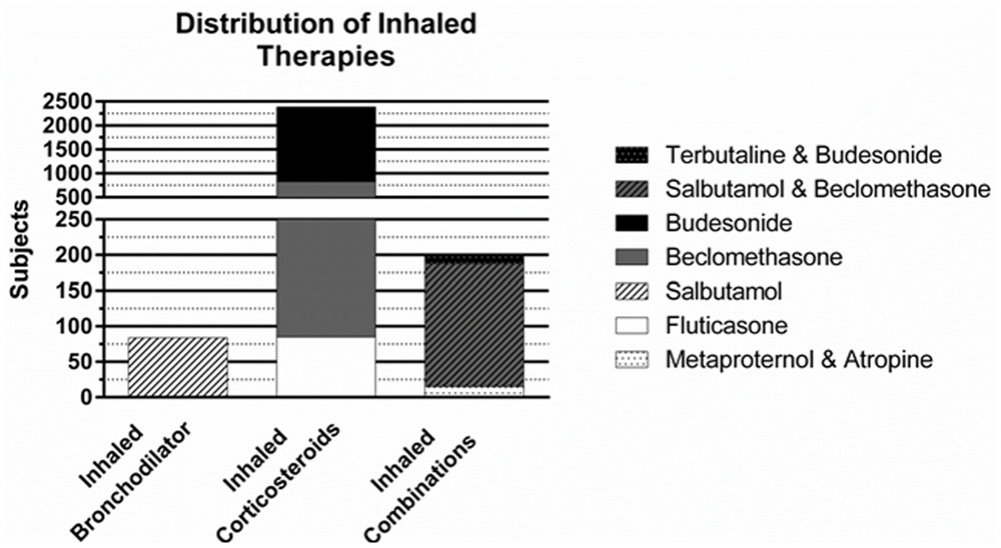
Distribution of Inhaled Medications. Distribution by drug classification of the inhaled therapies used among the 22 randomized control trials.

Fourteen studies evaluated ICS therapy (beclomethasone, budesonide, fluticasone) [22–35]. See Fig 2 for the medication distribution of the inhaled corticosteroids used. The combined characteristics of these studies are summarized in Table 3. They included 2383 subjects with a weighted GA of 26.2 (26–29) weeks, weighted BW of 853 (760–1114) grams, weighted PMA of 27.0 (26–31) weeks, and had a weighted age of 6 (0–45) days old at time of evaluation. Dosing regimens varied greatly as described in Table 1. Delivery methods included MDI and jet nebulizer to both mechanically ventilated and spontaneously breathing subjects.

The safety of inhaled beclomethasone was evaluated in 2 of the 14 studies [24, 29]. Each used an estimated MDI emitted dose of 1000 μg/kg/d using and assessed for the presence of adrenal suppression as a safety outcome marker. One study found no difference between plasma cortisol levels between the treatment and control groups and concluded that beclomethasone MDI can be delivered safely to neonates [29]. However, mildly reduced plasma cortisol levels were found in the other study, but they found no evidence of adrenal suppression during cosyntropin stimulation [24]. This study suggests further research is needed to clarify ICS therapy in neonates and such studies should focus on a variety of formulations, regimens, and delivery systems.

Therapeutic efficacy of ICS was assessed in 12 of the 14 studies [22, 23, 25–28, 30–35]. Two of these studies [32, 33] used a jet nebulizer, while the remaining utilized a MDI and holding chamber. Various outcome measure were assessed including changes in Crs, Rrs, inflammatory markers, and clinical outcomes such as duration of treatment, oxygen days, time to extubation, the development of BPD, length of stay, and death. Five of these studies compared ICS (budesonide [25, 31, 32] and beclomethasone [34, 35]) to systemically administered dexamethasone for infants with evolving BPD. Four of the studies included only mechanically ventilated subjects, [31, 32, 34, 35] whereas one study included both mechanically ventilated and spontaneously breathing subjects [26]. These studies revealed that ICS therapy had a slower onset of action, and was less effective in treating pulmonary inflammation as compared to systemic therapy. However, ICSs offered the benefit of fewer adverse side effects. The other seven studies evaluated the efficacy of ICS therapies (beclomethasone, [23, 30, 33] fluticasone, [26, 27] and budesonide [22, 28]) compared against inhaled placebo. Four of the studies included only mechanically ventilated subjects, [23, 27, 28, 30] whereas three studies included both mechanically ventilated and spontaneously breathing subjects [22, 26, 33]. In all but two studies [26, 28], subjects who were treated with ICS therapy exhibited improved outcomes over those treated with placebo. Noted improvements included earlier extubation [23, 27], reduced supplemental oxygen need [22], less systemic steroid use [23, 30], increased Crs[27, 33]and decreased Rrs [33], and reduced risk of BPD [22,30].

### Combination Therapies

Three studies evaluated combination therapies [36–38]. See Fig 2 for the medication distribution of the inhaled combination therapies used. The combined characteristics of these studies are summarized in Table 3. They included 198 subjects with a weighted GA of 27.8 (27–29) weeks, weighted BW of 1057 (898–1247) grams, weighted PMA of 30.7(29–45) weeks and had a weighted age of 20 (10 to 111) days old at time of evaluation. Beta agonist and ICS combinations were assessed in 2 of the 3 studies [36, 38]. The third study assessed the combination of a beta agonist and an anticholinergic [37]. Delivery methods included MDI for mechanically ventilated subjects, whereas MDI (mask with spacer) [36] and jet nebulizer (mask [37, 38] and/or hood [38]) were used for spontaneously breathing subjects.

In the first study evaluating the combination of a beta agonist and ICS (salbutamol, 1200 μg/kg/d and beclomethasone, 1000 μg/kg/d), the effectiveness of treatment was assessed using long-term outcomes such as BPD and death, and no significant differences were found between treatment groups and placebo [36]. The second beta agonist/ICS (terbutaline, 2 mg and budesonide, 0.5ml in 2ml of sodium chloride) combination study evaluated the delivery methods of aerosol hood and mask with a jet nebulizer [38]. In this study, patient tolerance and user preferences were assessed as primary outcomes. Secondary outcomes evaluated the efficacy of each delivery method. Results revealed that patients tolerated hood delivery better and users preferred this method over aerosol mask, additionally, the hood delivery was found to be at least as efficacious as the mask.

The remaining study assessed the combination of metaproterenol, 1 mg and atropine, 0.8 mg/kg [37]. This study used a jet nebulizer to deliver the combination therapy to spontaneously breathing subjects. Both metaproterenol and atropine individually had a positive effect on Rrs and maximum exhaled volume from functional residual capacity. However, there was no synergistic response when the drugs were combined.

## Discussion

Review of these 22 randomized control trials suggest flow obstruction associated with airway smooth muscle constriction and inflammation in infants on supplemental oxygen and mechanical ventilation can be alleviated through inhaled therapies. Evidence of such benefit is documented with improvements in Crs and Rrs following beta agonists, anticholinergics, and ICS therapies. However, clinically relevant long-term benefits such as reduced BPD, time to extubation, duration of supplemental oxygen, and length of stay remain unclear or absent. Additionally, variables such as age at study enrollment, point of BPD progression, and aerosol delivery methods, dose, etc. could potentially affect interpretation of study results.

This review is not without limitations. Limitations mostly involve adherence to strict inclusion criteria. Over 140 manuscripts were excluded for not meeting the minimum requirement of being randomized control design. Additionally, some studies could have been missed that were not indexed in the Academic Search Complete, CINAHL, PUBMED/MEDLINE, and Scopus databases. Including only studies of randomized control design, we attempted to minimize unmeasured bias; however, the quality assessment of data revealed an overall high risk of potential bias still remained.

Recent systematic reviews have provided insight to specific outcomes related to inhaled bronchodilator and ICS therapies in BPD, however, strict inclusion criteria of these reviews limit the ability to consider the complete body of work done in this area [10,11]. In order to meet our objective of identifying strengths and knowledge gaps in the literature regarding inhaled therapies in BPD and guide future research, we had to take a liberal approach and include within the analysis a wide range of outcomes, this created heterogeneity. Based on the National Academy of Medicine’s (formerly known as Institute of Medicine) guidelines [42], no quantitative synthesis (meta-analysis) was attempted due to the large degree of heterogeneity and quality assessment of included studies.

Despite a lack of evidence to support long-term use of bronchodilators, review of these randomized control studies has revealed that beta-agonists and anticholinergic therapies can acutely improve lung function in infants with evolving BPD [18–21, 37]. This is further supported in other observational studies [43–48]. Included in this review, Kao and colleagues (1989) used a jet nebulizer to deliver metaproterenol and atropine to fifteen spontaneously breathing subjects, nine of which remained on supplemental oxygen support [37]. Improvements in pulmonary function were noted by reduced Rrs and increased exhaled volume from functional residual capacity after administration of each inhaled medication independently. However, no synergistic effect was noted. Similarly, in an observational study DeBoeck and colleagues (1998) evaluated the combination of salbutamol and ipratropium bromide by jet nebulizer and face mask to twenty spontaneously breathing subjects with BPD [47]. Evaluating Rrs, they did not observe a synergistic benefit of combined therapy. However, Brundage and colleagues (1990) were able to achieve a synergistic effect when the dose of ipratropium bromide was increased above the standard dose [46]. Fok et al. [18] and Kao et al. [37] revealed through measurements of Rrs that the bronchodilator effect of salbutamol subsides after two and three hours respectively. Brundage et al. demonstrated that the synergistic effect of combined salbutamol and ipratropium bromide can promote a longer bronchodilator effect than each drug given independently [46]. They observed a continued effect at four hours post treatment. Due to a relatively short duration of bronchodilation with beta agonist therapy, further research is warranted to evaluate other combination therapies such as the long acting bronchodilator formoterol in combination with budesonide.

There are mixed results regarding the efficacy of ICS therapy in this review. Some studies suggest that they provide no benefit, [26, 28, 31, 32, 34, 35] whereas, others reveal ICS therapy can be safe and beneficial [22, 23, 27, 29 30, 33]. Studies do show that ICSs have a slower onset of action as compared to systemic steroids and can have similar side effects [24, 25, 31, 34, 35]. Careful review of the literature included in this paper suggests that ICS effectiveness may directly increase with prescribed dose. Seven ICS studies had prescribed doses of ≥ 1000 μg/day [23, 24, 27–30, 32]. Giep and colleagues (1996) found no short-term adverse effects at this dose level of MDI beclomethasone in nineteen mechanically ventilated subjects [29]. Three of these studies (beclomethasone [23, 30] & fluticasone [27]) that reported improved outcomes with ICS therapy had prescribed these higher doses [23, 24, 27, 30]. Two studies with no noted improvements at that ≥ 1000 μg/day dose level differed from the other studies [28, 32]. Kovács and colleagues (1998) used a jet nebulizer to deliver budesonide to mechanically ventilated subjects [32]. In comparison to other studies where improvements were noted, [23, 24, 27, 30] their study differed from others in both study medication and delivery method. In the remaining study that utilized a higher dose, but did not see improved outcomes, Merz and colleagues (1999) used a MDI, spacer and hand ventilation to deliver budesonide to mechanically ventilated subjects [28]. The duration of treatment in their study only lasted for 10 days, whereas, studies that showed improvements at this dose range reported treatment durations from 14 to 28 days in length [23, 24, 27, 30]. In regard to lower (<1000 μg/day) doses, one study (budesonide [22]) reported improved outcomes at a dose of 800 μg/day, whereas two studies (beclomethasone [34] & fluticasone [26]) report no benefit of ICS therapy with doses of 17 to 250 μg [26, 34]. Further research is needed to explore the safe and effective dosages in ICS therapy for infants with BPD. Despite having some evidence that ICS therapy can be beneficial in regards to improving outcomes such as duration of respiratory support, improvements in long-term outcomes of ICS therapy such as reduced length of stay, mortality, etc. are still lacking [22, 25–28, 31, 32, 35]. Denjean and colleagues (1998) in a randomized double blind trial evaluated combined salbutamol, 1200μg/d and beclomethasone, 1000μg/d [36]. They found no differences in development of BPD or mortality in a sample of 173 subjects [36]. In a recent trial, the largest to date (*n* = 863 infants), Bassler and colleagues (2015) aimed to maximize budesonide delivery to infants on CPAP and mechanical ventilation with a spacer device, in order to evaluate its effectiveness in reducing BPD and 18–22 month neurodevelopmental outcome [22, 49, 50]. They found no significant change in mortality following budesonide randomization at 12 hours of age until infants were either off supplementary oxygen and positive pressure support or had reached a gestational age of 32 0/7 weeks, but found a reduction of BPD at 36-weeks (relative risk 74 (0.60–0.91)) [22]. In addition to a large sample size, they conclude possible factors attributing to their positive findings could include a modest dose of ICS to augment lung deposition and early versus late initiation of therapy [22]. Additionally, they used a budesonide MDI formulation with valved holding chamber for delivery that was previously unavailable [22, 40, 51]. They were able to show a therapeutic benefit at a lower dose than previous studies [22]. Responses to therapies are dependent on the amount of aerosol delivered to the peripheral airways. Aerosol with a mass median aerodynamic diameter of 1–5 μm are capable of reaching the periphery [52]. Deposition can be effected by many variables such as aerosol composition, infant anatomy, the presence of an artificial airway, aerosol delivery devices, etc. [39–41, 52–55]. Due to the Montreal Protocol (1987), pharmaceutical companies were forced to develop MDIs with an environmental friendly propellant [56]. As a result, newly developed hydrofluoroalkane propelled formulations have a smaller mass median aerodynamic diameter as compared to traditional chlorofluorocarbon propelled devices [39–41]. The study conducted by Bassler et. al (2015) is the only study in this review conducted in the hydrofluoroalkane era [22].

There were multiple methods in which aerosols were delivered in this review. Four studies specifically evaluated delivery methods [17–19, 38]. Fok and colleagues (1996) used radiolabeled salbutamol and gamma imaging to evaluate two standardized delivery methods in spontaneously breathing and mechanically ventilated subjects [17]. Although lung deposition was small and variable, they found the MDI with a chamber spacing device placed inline to be a more efficacious method of delivery when compared to a jet nebulizer. However, Gappa and colleagues (1997) found the two devices to be equally efficient in delivery of salbutamol to spontaneously breathing subjects by measures of pulmonary mechanics [19]. Fok and colleagues (1998) again evaluated salbutamol delivery methods in a follow-up study of mechanically ventilated subjects [18]. The same two delivery methods were assessed as in their previous study, however, a third method; the ultrasonic nebulizer was added for evaluation. Findings revealed that the ultrasonic nebulizer out performed both the MDI and jet nebulizer with greater reductions in Rrs. Kugelman and colleagues (2006) evaluated terbutaline and budesonide combination therapy in spontaneously breathing subjects delivered by both hood and mask nebulization [38]. They concluded that hood nebulization was better tolerated by infants and was preferred over mask nebulization by users administering the aerosol treatments. Additionally, they concluded that hood nebulization was at least as effective as mask delivery based on respiratory assessment scores. Such scores rely on caregiver observations of retractions, assessment of breath sounds, and other measures that can be subjective and highly variable between scorers [57, 58]. Caution is necessary when drawing conclusions based on such observations. Future studies should focus on using clear objective physiological data when analyzing outcomes. Considering that there were only four studies [17–19, 38] with randomized design that evaluated the effectiveness of delivery methods to infants with BPD, it remains unclear which methods are optimal. Furthermore, these studies are now one to two decades old. Technology across this time frame has brought about many new products aimed at improving aerosol delivery. Specially designed masks with reduced dead space, innovative nebulizers, and redesigned MDI spacers are examples of new products that may prove beneficial in this select patient population [51, 59–62]. It is likely that the most optimal method of aerosol delivery will vary per the infant’s modality of respiratory support (e.g. infants on supplemental oxygen vs. nasal continuous positive airway pressure vs. mechanical ventilation) [51, 63–66]. Future research should focus on the evaluation of new and existing devices across different modalities of respiratory support. Additionally, new drugs and novel use of existing medications should be evaluated. Inhaled agents such as nitric oxide, furosemide, and pentoxifylline have been studied in infants with or at risk of developing BPD [67–69]. Due to a limited number of randomized control studies, no clear conclusions can be made regarding these novel drugs in infants with BPD.

Subjects’ ages at time of evaluation were well defined in all but one study in this review [33]. Based on the mean GA and age at time of evaluation, only one study evaluated inhaled medications in BPD beyond age corrected to term GA [37]. Given variability in age at time of evaluation, we should be cautious in making generalized statements that inhaled beta agonists, muscarinic antagonists, corticosteroids, or any combination of these therapies are ineffective in treating BPD. There are many variations in the clinical presentation and characteristics in infants who have evolving versus established BPD. Likewise, there are also many differences in degrees of BPD severity [14]. Since the majority of these past studies evaluated aerosol therapy in evolving BPD, it remains unclear whether or not such therapies are beneficial for post-term infants with chronic stable disease.

The pulmonary function measures used to assess response to therapy in this review are predominantly measures of Crs and Rrs. These measures can be highly variable and not as sensitive to changes in airway flow resistance [44, 47, 70]. “Adult-type” infant pulmonary function methods and equipment have become available for use infants since the studies included in this review, these tests are highly reproducible [71, 72]. De Boeck and colleagues (1998) noted that pulmonary function measures of forced flow may be a more sensitive and appropriate measures of airway response compared to Rrs [47]. Equally important, they concluded that not all infants with BPD respond to aerosol therapy, therefore, infants should be screened before starting treatments. Robin and colleagues (2004) assessed bronchodilator responsiveness in twenty-eight subjects with chronic stable BPD [73]. MDI albuterol was delivered by chamber and facemask to seventeen subjects who underwent pre and post bronchodilator response testing during infant pulmonary function testing. Only thirty five percent of patients had a significant positive response measured by forced expiratory flow analysis. In a longitudinal study evaluating lung volumes and forced flows by pulmonary function testing of forty four subjects with moderate to severe BPD at six, twelve, and twenty four months after discharge; Fakhourly and colleagues (2010) reported that response to bronchodilators was at thirty percent at six months post discharge and tapered to twenty percent by the end of the study at twenty four months post discharge [74]. This phenomenon could be related to tachyphylaxis, better control of airway hyper-reactivity though the use of controller medications such as ICS, or to new lung growth and healing associated with increasing age. These changes in response further suggest that children with BPD have a variable response to inhaled medications, which could be related to disease progression and severity. Since bronchodilators are short-acting medications and used as rescue medications in obstructive lung diseases as opposed to controller medications that aim to improve longer-term outcomes, future studies of their effectiveness in bronchodilator responders with BPD might consider physiologic symptom relief and patient comfort as a relevant and important patient-centered outcome.

Considering only approximately thirty percent of children with BPD are responders to bronchodilators, [73, 74] subjects should be carefully screened for responsiveness before being treated or enrolled in research evaluating the outcomes of such therapies [75]. We should be hesitant of past studies where subjects were treated without being screened for responsiveness. Inclusion of non-responders in treatments groups could potentially mask benefits of therapy in true responders and affect the outcome and interpretation of results. Future research should focus on evaluating bronchodilator/ICS therapies and their effect on long-term outcomes in subjects who are known responders to such therapies. This can be accomplished by utilizing infant pulmonary function testing methodology with a bronchodilator challenge [72, 76]. This would allow researchers to stratify subjects into responder and non-responder groups. Additional repeated measures after the initiation of ICS therapies could provide valuable insight to those with flow obstruction due to reactive airways and inflammation.

## Summary

In summary, this review reports on twenty-two randomized control trials pertaining to the use of inhaled beta agonists, anticholinergics, and corticosteroids in infants with BPD. Although these inhaled therapies seem to have some benefit, there is very limited data to suggest these treatments improve long-term outcomes in infants with BPD. The majority of existing literature focuses on evolving BPD. Further research is needed to evaluate bronchodilator use in infants with chronic stable BPD. New inhaled drugs and devices have been developed since the publication of much of the literature included in this review. Future studies should evaluate new drug combinations and devices as compared to traditional methods. These studies should aim to find the optimal method of delivery across all modalities of respiratory support. Finally, infants should be stratified into responder and non-responder groups so that potential benefits of therapy are not masked by non-responders.

## Supporting Information

S1 File PRISMA Checklist. (DOC)

## Abbreviations

BPD: bronchopulmonary dysplasia
BW: birth weight
C_L, dyn_: lung compliance
Crs: respiratory system compliance
GA: gestational age
ICS: inhaled corticosteroids
MDI: metered dose
R_L, dyn_: lung resistance
Rrs: respiratory system resistance
